# Molecular targeted therapies in head and neck cancer - An update of recent developements -

**DOI:** 10.1186/1758-3284-2-8

**Published:** 2010-04-14

**Authors:** Martin Goerner, Tanguy Y Seiwert, Holger Sudhoff

**Affiliations:** 1 Community Hospital Bielefeld, Department of Hematology, Oncology and Palliative Care, Teutoburger Str. 60, 33604 Bielefeld, Germany; 2 The University of Chicago, Section of Hematology/Oncology, 5841 S Maryland Ave, MC2115 Chicago, IL, USA; 3 Community Hospital Bielefeld, Department of Otolaryngology, Teutoburger Str. 60, 33604 Bielefeld, Germany

## Abstract

Targeted therapies have made their way into clinical practice during the past decade. They have caused a major impact on the survival of cancer patients in many areas of clinical oncology and hematology. Indeed, in some hematologic malignancies, such as chronic myelogenous leukemia or non-Hodgkin's lymphomas, biologicals and antibodies specifically designed to target tumour-specific proteins have revolutionized treatment standards. In solid tumours, new drugs targeting EGF- or VEGF- receptors are now approved and are entering clinical practise for treatment of colon, lung, kidney and other cancers, either alone or in combination with conventional treatment approaches.

Recent data have now shown that molecular targeted therapy might display efficacy in patients with head and neck squamous cell carcinoma (HNSCC) as well. The evaluated biologicals are generally well tolerated from HNSCC patients, who usually have the burden of multiple co-morbidities that interfere with conventional systemic treatment options. Therefore, molecular targeted therapies offer new treatment options even for heavily pretreated and seriously ill patients usually unable to tolerate chemotherapy or radiation therapy.

The two most promising and advanced strategies are the blockage of growth-factor based cellular signalling and interference with angiogenesis-related pathways. But inhibitors of alternative targets, such as Scr and proteasomes, have already been evaluated in early clinical trials with HNSCC patients.

## Introduction

Squamous cell carcinoma of the head and neck (SCCHN) represents the eighth leading cause of cancer worldwide. Despite recent advances in surgery and radiotherapy, overall cure is achieved in less than 50% of patients. In contrast to many other cancers, distant metastases are rarely present at diagnosis, but due to better local control, the incidence of systemic spread is rapidly increasing. Those with recurrent or metastatic disease have a poor prognosis, with median survival rates of 6-10 months [1]. Systemic chemotherapy remains the only effective treatment option, but it is associated with significant toxicity rates in HNSCC patients, who usually have a high prevalence of co-morbidities and problematic lifestyle habits [2]. Therefore, additional treatment options that have the potential to improve outcome and that show a toxicity profile different from cytotoxic agents are desperately needed to complement presently available treatment tools.

New agents that specifically target cellular pathways associated with carcinogenesis are promising candidates, because they are already successfully used in other hematological malignancies as well as in solid tumours, such as colorectal or lung cancer [3].

Two primary strategies that might have the potential to change clinical routine within the near future will be discussed in this review: first, blocking epidermal growth factor-based cellular signalling (EGFR-associated) and second, blocking angiogenesis related cellular signalling (VEGFR-associated). In addition, we will review data on new drugs that target molecular targets other than EGFR and VEGF and discuss their relevance for HNSCC treatment.

## The role of EGF-R signalling in HNSCC

The EGF-R is a member of the human epidermal receptor (HER)/Erb-B family, a group of tyrosine kinases that transduce extracellular signals to intracellular responses influencing cell proliferation, apoptosis, angiogenesis, and the capacity of tumour cells to metastasize [4]. It has been shown that EGF-R and TGF-α, one of the seven known ligands of EGF-R, are overexpressed in many solid tumours, including colorectal cancer, NSCLC, and HNSCC [5]. Furthermore, EGF-R-overexpression as well as increased m-RNA levels of TGF-α in tumours are usually associated with poorer responses to radiotherapy and have been shown to be strong predictors of decreased disease-free survival [6]. These observations are the rationale for the development of EGF-R-targeted therapies, which are intended to interrupt EGF-R-mediated pathways.

Among EGF-R-targeting therapies, there are two large categories of molecules: monoclonal antibodies, which recognize the ligand-binding domain and interfere with receptor activation, and tyrosine kinase inhibitors which bind to the cytoplasmatic region and influence with downstream signalling events.

### Anti-EGF-R antibodies

Cetuximab is a chimeric human/murine monoclonal antibody of the IgG1 isotype that binds to the EGF-R with a higher affinity than its endogenous ligands, preventing dimerization, internalisation and autophosphorylation. Preclinical studies show at least three different mechanisms by which cetuximab affects tumour cells. First, it enhances tumour-cell apoptosis and inhibits proliferation as well as invasiveness by blocking the tyrosine-kinase-mediated pathways. Second, antibody-dependent cell-mediated toxicity, which is associated specifically with the IgG1 isotype, contributes to the anticancer activity. Finally, cetuximab may block the nuclear import of EGF-R, preventing activation of the DNA repair mechanism that protects cells from radiation- or chemotherapy-induced DNA damage [7-9].

Two other anti-EGF-R MoABs are currently tested in large clinical trials. Panitumumab is a fully human, IgG2 EGF-R-targeting antibody that is already approved for metastatic colon cancer and is tested in locally advanced disease in combination with radiotherapy[10]. Zalutumumab, also a fully human antibody of the IgG1 type, is currently being evaluated in a randomized phase III trial concerning best supportive care for advanced platinum refractory patients [11-14]. While the use of these both agents remains experimental until study results are published, profound clinical data are available for cetuximab, both in the adjuvant and palliative setting.

#### Cetuximab in locally advanced HNSCC

Cetuximab is approved in combination with irradiation in locally advanced disease based on a multinational, randomized phase III trial comparing radiotherapy plus cetuximab with radiotherapy alone. Results published by Bonner in 2006 demonstrated significantly prolonged locoregional control and overall survival without adversely affecting quality of life[15]. Risk of locoregional failure was significantly reduced, resulting in a 9-month increase in median locoregional control. Median overall survival could be prolonged to a median of 49 months (vs. 29 months). In addition, preservation of larynx function, which is a major determinant of life quality, seemed to be better in the cetuximab arm. As expected from the experiences in other malignancies, acneiforme rash and infusion-related reactions were the only reported toxicities [16]. Although no data are available from studies comparing cetuximab plus radiotherapy to the standard treatment of platin-based radio-chemotherapy, this regimen has to be considered an important alternative, particularly for patients in poor medical condition.

#### Cetuximab in metastatic HNSCC

In previously untreated patients with metastatic HNSCC, cisplatin-based chemotherapy is considered standard. This approach is now challenged by the recently published results of the EXTREME study (**E**rbitux in First-line **T**reatment of **R**ecurrent or **Me**tastatic Head and Neck Cancer). In this controlled randomized phase III trial, 442 patients who were not amenable to local therapy and had not received any systemic treatment received either cisplatin or carboplatin, together with 5-fluorouracil or a combination of this chemotherapy with cetuximab. Preliminary data demonstrated median survival times that differed significantly between the two study arms. The addition of cetuximab prolonged OS from 7.4 months to 10.1 months and disease-free survival from 3.3 to 5.6 months [17]. Although these data support the use of cetuximab in first-line combinations, still many patients receive platin-containing chemotherapy combinations without cetuximab up front. In case of recurrence, cetuximab monotherapy might then offer a second-line option with significant antitumour activity to these platin-resistant patients. In a retrospective analysis Vermorken et al. found an absolute increase of 2.5 months in median overall survival in cetuximab-treated patients compared to historical controls treated with best supportive care only[18]. In general, response rates to cetuximab in studies examining this pretreated patient population usually are in the range of 6-20%. Interestingly, no further benefit could be achieved adding cisplatin to cetuximab [19].

### EGF-R-targeted tyrosine-kinase inhibitors

TKIs bind intracellularly to EGF-R tyrosine-kinase and block downstream signalling pathways. Gefitinib and erlotinib, both administered orally once a day, are the two most advanced TKIs and are both approved for certain indications in non-small cell lung cancer. They have been evaluated in phase I/II trials as monotherapies in recurrent or metastatic HNSCC with response rates of 4-10%. Unfortunately, the only available phase III study involving 486 patients with recurrent HNSCC reported no improvement in response rates and overall length of survival with the addition of gefitinib at different dosing schedules to methotrexate when compared to methotrexate treatment alone [20].

Other studies are now evaluating gefitinib and erlotinib in combination with more aggressive chemotherapy regimens, such as platinum or docetaxel, and either with or without concurrent radiotherapy. Until these data are available, the use of gefitinib and erlotinib remains experimental, and important questions have to be answered before clinical use can be recommended.

### EGF-R and HER-2 combined targeted tyrosine-kinase inhibitors

HER-2 has also been found to be expressed in a significant proportion of EGF-R-positive HNSCCs. Since EGF-R and HER-2 heterodimerize to form functional signalling complexes, tyrosine-kinase inhibitors with dual specificity against HER-2 and EGF-R, such as lapatinib, have been investigated in phase I and II studies. In one of these studies lapatinib, which is already approved for breast cancer treatment, showed disease stabilization rates of about 20% in patients pretreated with anti-EGFR compounds[21], and therefore its efficacy in the adjuvant setting is currently being explored in ongoing phase III studies. Furthermore the irreversible EGFR/her-2 inhibitor BIBW-2992 is being compared head to head with cetuximab in a randomized crossover study. Due to its irreversible inhibition BIBW-2992 remains active in many EGF-R mutations, including the EGF-RvIII mutation, which has been reported in HNSCC. The study completed enrollment in 2009 and initial results are anticipated for 2010.

## The role of angiogenesis in HNSCC

Similar to other solid tumours, angiogenesis plays an important role in the pathogenesis of HNSCC. Vascular endothelial growth factors (VEGF) and its receptors are expressed in most cases of HNSCC, and multiple preclinical studies have shown that these markers are associated with tumour progression, changes in microvessel density, and development of lymph node metastasis. In addition, increased levels of VEGF in serum of patients with HNSCC appear to induce tumour growth, metastasis, and treatment failure [22]. Nude mice experiments have shown that inhibition of VEGF pathways markedly decreases angiogenesis and tumour growth [23]. Therefore several strategies to target VEGF-mediated angiogenesis have been developed and are currently being explored in clinical trials.

### VEGF ligand targeted therapy

Bevacicumab is a fully humanized monoclonal antibody binding VEGF with proven activity in colorectal, breast, and non-small cell lung cancer. In HNSCC, bevacicumab shows little single agent activity, but a small phase I/II study in combination with erlotinib in metastatic or incurable recurrent disease showed an overall response rate of about 15% and a median survival of 7.1 months. In general the regimen was well tolerated, with rash, diarrhea, and fatigue as the predominant side effects. But as in other entities, a small but significantly increased risk of bevacicumab-associated bleeding events have been reported in addition to other, more easily manageable side effects, such as hypertension and fluid retention, in this trial [24]. In several ongoing trials bevacicumab is currently being explored in combination with chemotherapy, radiation therapy, or EGFR inhibitors, but so far no clinical data are available to recommend the use of bevacicumab in the clinical routine.

### Small molecules targeting VEGF-receptor

Sorafenib and sunitinib are multikinase inhibitors that are already approved for several other cancer types and have shown their ability to inhibit the intracellular activity of VEGF-R and to block downstream signalling. Promising early clinical results were obtained in a small trial in refractory or metastasizing HNSCC patients, with single agent sorafenib achieving stable disease in 10/26 patients and a median overall survival of 8 months [25]. A non-randomized phase II trial evaluating a combination of paclitaxel, carboplatin, and sorafenib is currently ongoing. Sunitinib, which is approved for gastrointestinal stromal tumour and renal cell carcinoma, as well as vandetanib, a selective dual inhibitor of EGFR and VEGF pathways, are currently being evaluated in phase II trials, either alone or in combination with cytotoxic chemotherapy in advanced HNSCC.

## Other potential targets

Src kinases are involved in the regulation of a variety of normal cellular signal transduction pathways, and they influence cell proliferation, survival, angiogenesis, migration, and adhesion. In general, levels of Src expression or activation in epithelial tumours correlate with disease progression. It is important that Src activation results in potentiation of EGF-R-mediated tumour growth by stimulating the same downstream pathways like FAK, STAT, and PI3K[26]. In fact, recently published in vitro experiments show that Src family kinases are highly activated in cetuximab-resistant cells and that they enhance EGF-R activation despite the cetuximab-bound receptor. In these experiments inhibition of Src kinases in originally cetuximab-resistant cell lines resulted in a regaining of sensitivity against cetuximab, indicating a close interaction between Src and EGF-R regarding the processes causing cetuximab resistance in tumour cells [27].

Dual targeted treatment approaches directed at both EGF-R and Src might, therefore, be a feasible strategy for overcoming or preventing acquired resistance to cetuximab.

Dasatinib is a potent inhibitor of multiple oncogenic kinases including Src, cKIT, BCR-ABL, PDGFR, and ephrin A. Because of its ability to inhibit BCR-ABL, it was approved for treatment of chronic myeloid leukemia in 2006. Currently dasatinib is being evaluated in phase I clinical trials for solid tumours either alone or in combination with cetuximab [28].

*Proteasomes *are proteinases, which play a critical role in degradation of the proteins responsible for the control of cell growth. Inhibitors of proteasomes have demonstrated antitumour effects associated with the induction of apoptosis and sensitization of malignant cells to conventional cytotoxic drugs. Bortezomib, a small molecule inhibitor of proteasomes, has shown efficacy against myeloma and lymphomas, and was recently tested in phase II trials in HNSCC in combination with docetaxel or irinotecan, respectively[29]. Preliminary results have shown 50% disease control rates in recurrent or metastasizing HNSCC patients with the use of low-dose bortezomib[30]. Phase III trials with optimized dosing schedules are needed to confirm these promising new treatment options, which were usually associated with acceptable toxicity.

## Conclusion

Molecular targeted therapies are promising novel treatment options for patients with HNSCC. While EGFR-targeting approaches have shown significant but limited efficacy and are already approved for treatment in advanced HNSCC, other options, such as inhibitors of antiangiogenesis, proteasomes, or multifunctional tyrosine kinases are currently evaluated in phase I or II studies, either as single agent treatment or in combination with conventional cytotoxic drugs. Though multiple questions regarding dosing, combination and patient selection need to be answered, molecular targeted treatment will complement conventional chemo- and radiation therapy in patients with HNSCC in the near future. Especially the low toxicity profiles of these new agents are very promising. So far however, all molecular targeted therapies with the exception of cetuximab should be used in the context of clinical trials only.

## Competing interests

The authors declare that they have no competing interests.

## Authors' contributions

MG and HS performed the literature research and composed the manuscript.

TS critically revised the manuscript. All authors approved the final version of the manuscript.
